# Interpretable QSPR Modeling of Bipolar Disorder Drugs Using Topological Indices and Machine Learning Regression

**DOI:** 10.1021/acsomega.6c06753

**Published:** 2026-08-27

**Authors:** Özge Çolakoğlu, Anthuvan Joseph Benjamin

**Affiliations:** † Department of Mathematics, Science Faculty, Mersin University, Mersin 33343, Turkey; ‡ Department of Mathematics “Tullio Levi-Civita”, 9308 University of Padua, Padua 35121, Italy

## Abstract

The increasing demand for efficient and interpretable computational methods for molecular property prediction has stimulated the development of quantitative structure–property relationship (QSPR) models based on graph-theoretical molecular descriptors. Topological indices are numerical descriptors derived from graph-theoretical representations of chemical structures and are widely used in QSPR studies to predict physicochemical and biological properties. In this study, degree-based and neighborhood-degree-sum-based topological indices were computed for 18 drugs used to treat bipolar disorder and employed as descriptors for modeling physicochemical properties. linear regression, ridge regression, random forest, and XGBoost were systematically evaluated to investigate the suitability of classical and machine learning regression methods for QSPR modeling of a small molecular data set. The comparative analysis demonstrates that although ensemble machine-learning models (random forest and XGBoost) were capable of capturing nonlinear relationships, their predictive performance deteriorated under leave-one-out cross-validation because of the limited sample size. In contrast, ridge regression consistently produced the lowest prediction errors while preserving interpretability. Neighborhood-degree-sum-based descriptors produced lower prediction errors for seven of the nine investigated physicochemical properties, whereas degree-based descriptors remained stronger for boiling point and heavy atom count. Although meaningful relationships between molecular topology and physicochemical properties were identified, the findings are limited by the small data set and lack of external validation. Therefore, the proposed models should be considered as exploratory approximations. This study provides new insights into the structure–property relationships of bipolar disorder drugs and establishes a foundation for future studies using larger molecular data sets.

## Graphical abstract

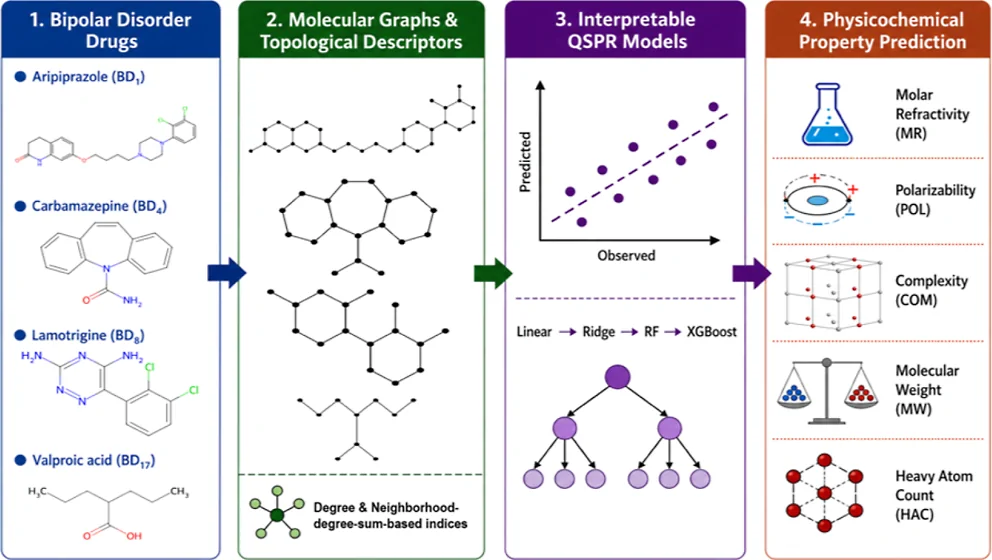

## Introduction

Graph theory provides a strong framework for representing chemical structures by using molecular graphs, where vertices represent non-hydrogen atoms and edges represent chemical bonds. Numerical descriptors derived from the topology of these graphs are known as topological indices. While these indices do not directly calculate physicochemical properties, they serve as essential components for simplified, interpretable, and computationally efficient models. Specifically, topological indices offer a cost-effective alternative to full quantum chemical calculations by effectively capturing fundamental structural information. Topological indices can be utilized to predict the physicochemical and biological characteristics of various compounds in quantitative structure–property relationship (QSPR) and quantitative structure–activity relationship (QSAR) studies.^1^ Wiener’s pioneering research^2^ initially employed structural descriptors to forecast alkaline properties. Topological indices can generally be classified into degree-based, neighborhood-based, distance-based, and eigenvalues-based. Degree-based indices characterize local connectivity through vertex degrees,^3^ whereas neighborhood-degree-sum indices incorporate information from adjacent vertices.^4^ In contrast, degree-based indices account for atomic valence, while distance-based indices primarily quantify global molecular topology.^5,6^ A graph derived from the eigenvalues of another graph holds a significant place in the literature for energy-related studies.^7^ Polynomials can be used to calculate the index of a graph. Polynomials (M-polynomial, NM-polynomial, etc.) can facilitate the calculation and interpretation of large molecules.^8,9^ However, the method of calculating index values using edge partitioning provides an efficient tool for calculating suitable index values used in QSPR regression models. There are many studies in the literature on topological indices and QSPR/QSAR research and, more recently, on machine learning in calculating the properties of chemical structures.^10–13^ Qin et al. investigated graph theory and machine learning for predicting the properties of bladder cancer drugs.^14^ Hanif et al.^15^ obtained a model for predicting some properties of amphetamine-type drugs using NM-topological indices and machine learning. Bayati et al.^16^ modeled equations for predicting asthma drug properties via machine learning and indices. Ejima et al.^17^ studied the properties of mental disorder drugs with machine learning and topological indices. Fazal et al.^18^ predicted the properties of colorectal cancer drugs using curvilinear regression via machine learning. Koam et al.^19^ used machine learning to predict the properties of polycyclic aromatic hydrocarbons. Abubakar et al.^20^ studied the prediction of antibacterial drug properties using indices and machine learning. Narmatov et al. used machine learning to obtain information about the entropy and energy of chemicals from small (bio)­chemical data sets.^21^

Bipolar disorder, a persistent mental health ailment, is distinguished by recurrent episodes of mania and depression and is generally categorized into bipolar I and bipolar II disorders, contingent upon the intensity and frequency of mood episodes.^22^ While the precise cause of the disorder is not fully understood, effective treatment is achievable through pharmacological approaches, encompassing mood stabilizers like lithium and valproate, in addition to antipsychotic drugs.^23^ Despite the existence of sophisticated cheminformatics tools capable of directly calculating physicochemical properties, the demand persists for models that are simplified, easily understood, and computationally efficient. In this regard, topological indices offer a cost-effective alternative, encapsulating crucial structural characteristics without requiring computationally intensive quantum chemical calculations.

This study aims to assess how well degree-based and neighborhood-degree-sum-based topological indices can predict the important physicochemical properties of drugs used to treat bipolar disorder. To achieve this, several regression models were developed and evaluated, including linear regression, ridge regression, random forest, and XGBoost. Random forest and XGBoost were included to assess whether increased model complexity provides predictive advantages over classical regression for small data sets. These models were assessed using leave-one-out cross-validation, which is useful when the sample size is small. This study makes three primary contributions: First, it provides a systematic comparison between degree-based and neighborhood-degree-sum-based topological descriptors for the QSPR modeling of bipolar disorder drugs. Second, it evaluates interpretable regression and machine learning regression models using a rigorous LOOCV framework for small data sets. Third, it identifies the model complexity that best balances predictive performance and interpretability for small data sets. Unlike many previous studies that prioritize how well predictions match reality, this research emphasizes understanding the relationships between structures and properties. Through a focused study of bipolar disorder drugs, this work provides insights into descriptor selection and the applicability of graph-theoretical indices for small-sample QSPR modeling.

## Materials and Methods

Let Γ­(*V*(Γ), *E*(Γ)) be a graph. Let *d*_τ_ denote the degree of a vertex τ in a graph Γ. *N*(τ) is the set of the neighbors of τ ∈ *V*(Γ).^1^

Assume that $\varsigma_{ij}=\left\{\left|E_{\left(i,j\right)}\left(\Gamma \right)\right|,\left(d_{\tau },d_{q}\right)=\left(i,j\right),\text{for}\;\tau q\in E\left(\Gamma \right)\right\}$ and *S*_τ_ = ∑_x∈*N*(τ)_ *d*_*x*_

$$\varsigma_{ij}^{*}=\left\{\left|NE_{\left(i,j\right)}\left(\Gamma \right)\right|,\left(S_{\tau },S_{q}\right)=\left(i,j\right),\text{for}\;\tau q\in E\left(\Gamma \right)\right\}$$

Table 1 lists the mathematical expressions of the degree-based topological indices, namely, the first Zagreb index (*M*_1_(Γ)), second Zagreb index (*M*_2_(Γ)), forgotten index (*F*(Γ)), symmetric division degree index (*SDD*(Γ)), inverse sum indeg index (*ISI*(Γ)), harmonic index (*H*(Γ)), sum connectivity index (*SC*(Γ)), Randić index (*R*(Γ)), sigma index (σ­(Γ)), Sombor index (*SO*(Γ)), atom–bond connectivity index (*ABC*(Γ)), atom–bond sum-connectivity index (*ABS*(Γ)), and augmented Zagreb index (*AZ*(Γ)). Replacing the vertex degree *d*_τ_ by the neighborhood-degree-sum *S*_τ_ in these definitions yields their corresponding neighborhood versions, denoted by *M*_1_^*^(Γ), *M*_2_^*^(Γ), F*­(Γ), SDD*­(Γ), ISI*­(Γ), H*­(Γ), SC*­(Γ), R*­(Γ), σ*­(Γ), SO*­(Γ), ABC*­(Γ), ABS*­(Γ), and AZ*­(Γ), which are respectively known as the third Zagreb index,^34^ neighborhood second Zagreb index,^35^ neighborhood forgotten index,^35^ fifth NDe index,^36^ neighborhood inverse sum index,^37^ neighborhood harmonic index,^37^ neighborhood sum connectivity index,^36,38^ general Randić index,^37^ neighborhood sigma index, neighborhood Sombor index,^39^ neighborhood atom–bond connectivity index,^40^ neighborhood atom–bond sum-connectivity index,^41^ and Sanskruti index.^42^

**Table 1:** Various Degree-Based Topological Indices

topological index	mathematical expression
first Zagreb index^1^	*M*_1_(Γ) = ∑_τ*q*∈*E*(Γ)_ (*d*_τ_ + *d*_*q*_)
second Zagreb index^1^	*M*_2_(Γ) = ∑_τ*q*∈*E*(Γ)_ *d*_τ_ *d*_*q*_
forgotten index^24^	$F\left(\Gamma \right)=\sum_{\tau q\in E\left(\Gamma \right)}\left(d_{\tau }^{2}+d_{q}^{2}\right)$
symmetric division degree index^25^	$SDD\left(\Gamma \right)=\sum_{\tau q\in E\left(\Gamma \right)}\frac{d_{\tau }^{2}+d_{q}^{2}}{d_{\tau }d_{q}}$
inverse sum indeg index^25^	$ISI\left(\Gamma \right)=\sum_{\tau q\in E\left(\Gamma \right)}\frac{d_{\tau }d_{q}}{d_{\tau }+d_{q}}$
harmonic index^26^	$H\left(\Gamma \right)=\sum_{\tau q\in E\left(\Gamma \right)}\frac{2}{d_{\tau }+d_{q}}$
sum connectivity index^27^	$SC\left(\Gamma \right)=\sum_{\tau q\in E\left(\Gamma \right)}\frac{1}{\sqrt{d_{\tau }+d_{q}}}$
Randić index^28^	$R\left(\Gamma \right)=\sum_{\tau q\in E\left(\Gamma \right)}\frac{1}{\sqrt{d_{\tau }d_{q}}}$
sigma index^29^	$\sigma \left(\Gamma \right)=\sum_{\tau q\in E\left(\Gamma \right)}{\left(d_{\tau }-d_{q}\right)}^{2}$
Sombor index^30^	$SO\left(\Gamma \right)=\sum_{\tau q\in E\left(\Gamma \right)}\sqrt{d_{\tau }^{2}+d_{q}^{2}}$
atom-bond connectivity index^31^	$ABC\left(\Gamma \right)=\sum_{\tau q\in E\left(\Gamma \right)}\sqrt{\frac{d_{\tau }+d_{q}-2}{d_{\tau }d_{q}}}$
atom-bond sum-connectivity index^32^	$ABS\left(\Gamma \right)=\sum_{\tau q\in E\left(\Gamma \right)}\sqrt{\frac{d_{\tau }+d_{q}-2}{d_{\tau }+d_{q}}}$
augmented Zagreb index^33^	$AZ\left(\Gamma \right)=\sum_{\tau q\in E\left(\Gamma \right)}{\left(\frac{d_{\tau }d_{q}}{d_{\tau }+d_{q}-2}\right)}^{3}$

There are numerous studies on calculating chemical structure indices based on the sums of degrees and the degrees of neighboring vertices. Mondal et al. investigated the NM polynomial and degrees of crystallographic structures.^43^ Verma et al. defined NM-polynomials and their indices and also performed calculations for bismuth tri-iodide-type graphs.^44^ Kirmani et al. studied these indices for graph models of drugs used to treat COVID-19.^45^

Some drugs used in the treatment of bipolar disorder are aripiprazole (BD_1_), aripiprazole lauroxil (BD_2_), asenapine (BD_3_), carbamazepine (BD_4_), cariprazine (BD_5_), clozapine (BD_6_), haloperidol (BD_7_), lamotrigine (BD_8_), lumateperone (BD_9_), lurasidone (BD_10_), olanzapine (BD_11_), oxcarbazepine (BD_12_), paliperidone (BD_13_), paroxetine (BD_14_), quetiapine (BD_15_), riseridone (BD_16_), valproic acid (BD_17_), and ziprasidone (BD_18_), and their chemical structures are given in Figure 1. Note that the symbols in parentheses next to the drug names represent the molecular graphs of the drugs.

**Figure 1:**
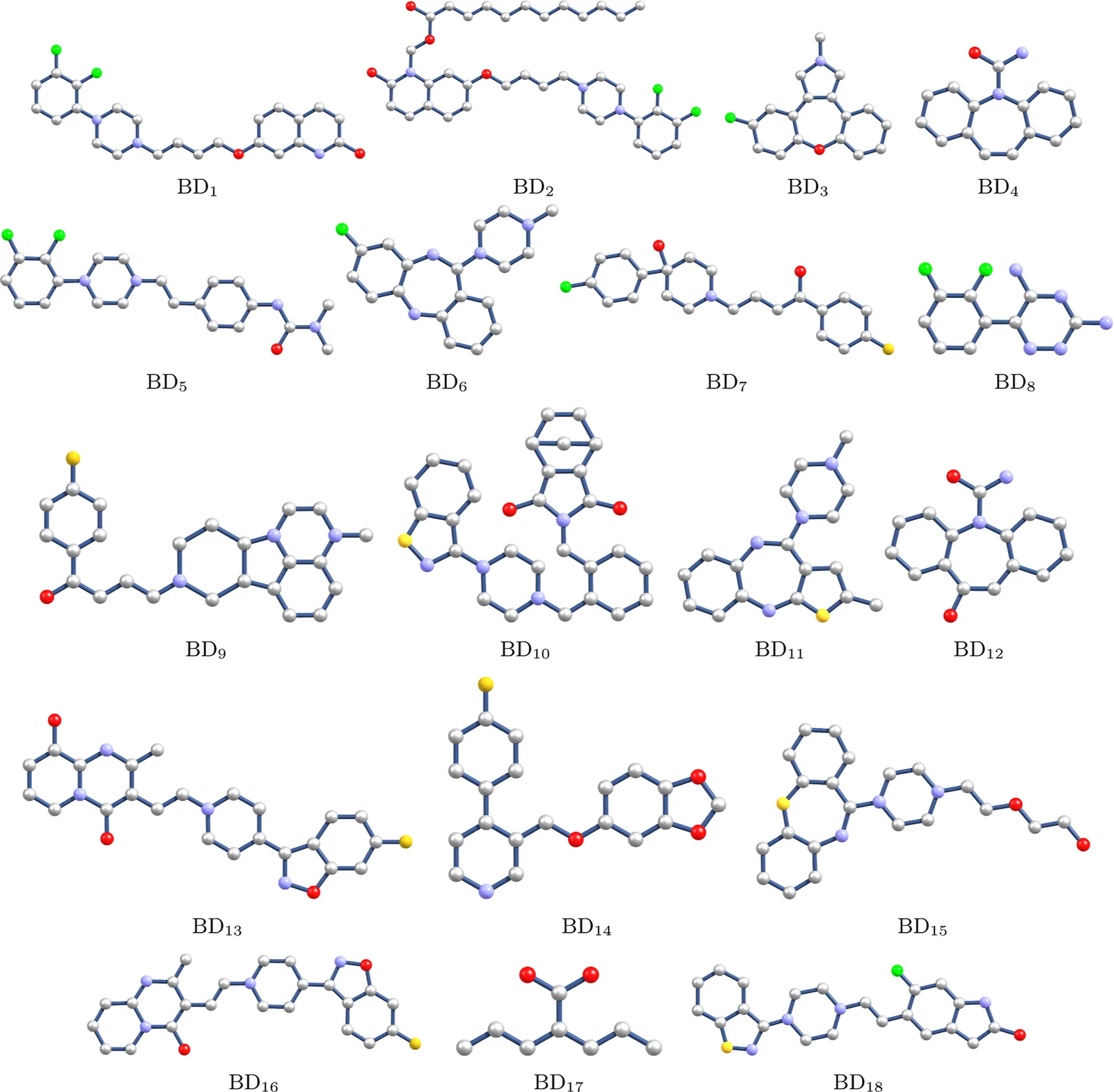
Chemical structures of drugs used in the treatment of bipolar disorder.

Table 2 summarizes the physicochemical properties of selected drugs used in the treatment of bipolar disorder, including boiling point (BP, °C at 760 mmHg), enthalpy of vaporization (EoV, kJ mol^–1^), flash point (FP, °C), molar refractivity (MR, cm^3^), polarizability (POL, 10^–24^ cm^3^), molar volume (MV, cm^3^), molecular weight (MW, g mol^–1^), molecular complexity (COM), and heavy atom count (HAC). The benchmark data sets comprising the chemical structures and physicochemical properties of these compounds are obtained from PubChem^46^ and ChemSpider.^47^ It is important to note that the objective of this study is not to replace existing cheminformatics tools, but rather to explore whether graph-theoretical descriptors can serve as surrogate predictors and provide interpretable relationships between molecular structures and physicochemical properties. Such approaches are particularly useful in early-stage screening or in environments where computational resources are limited. The data set comprises all bipolar disorder drugs for which consistent molecular structures and the selected physicochemical properties were simultaneously available from the considered public databases. Although relatively small, this data set provides a suitable benchmark for exploratory QSPR modeling of bipolar disorder drugs.

**Table 2:** Properties of Bipolar Disorder Drugs

BDs	BP	EoV	FP	MR	POL	MV	MW	COM	HAC
BD_1_	646.2	95.3	344.6	120.3	47.7	355	448.4	559	30
BD_2_	781.7	113.7	426.6	182.6	72.4	572.7	660.7	858	45
BD_3_	357.9	60.3	170.2	80.5	31.9	232.1	285.8	363	20
BD_4_	411	66.3	202.4	60.7	27.6	186.6	236.27	326	18
BD_5_	600.1	89.3	316.7	117.2	46.4	344.5	427.4	491	28
BD_6_	489.2	75.5	249.6	93.7	37.2	247.7	326.8	446	23
BD_7_	529	84.6	273.8	101	40	303.3	375.9	451	26
BD_8_	503.1	77.2	258.1	63.4	25.1	162.9	256.09	242	16
BD_9_	556.4	83.8	290.3	112.7	44.7	310.4	393.5	593	29
BD_10_	623.4	92.3	330.8	138.8	55	386.8	492.7	804	35
BD_11_	476	74	241.7	92.2	36.5	236	312.4	432	22
BD_12_	457.2	71.7	230.3	70.2	27.8	189.8	252.27	382	19
BD_13_	612.3	95.6	324.1	112.6	44.6	294.1	426.5	764	31
BD_14_	451.7	71.1	227	87.9	34.9	271.5	329.4	402	24
BD_15_	556.5	88.2	290.4	110.2	43.7	301.1	383.5	496	27
BD_16_	572.4	85.8	300	111.7	44.3	296.8	410.5	731	30
BD_17_	220	50.3	111.1	40.6	16.1	155.6	144.21	93.4	10
BD_18_	554.8	83.6	289.3	114.1	45.2	301.4	412.9	573	28

### Modeling Approach

In this study, various regression methodologies are evaluated to establish quantitative structure–property relationships (QSPR) using degree-based and neighborhood-degree-sum topological indices. Given the small sample sizes (*n* ≤ 50), model selection and validation techniques are implemented to maximize robustness and generalizability. In this study, linear, ridge, random forest, and XGBoost regression models are studied.

#### Linear Regression

Linear regression is employed to model the dependence of a target property *P* on a single topological index *D*_*i*_. In this approach, a linear relationship between the dependent and independent variables is assumed, expressed as *P* = γ_0_ + γ_1_ *D*_*i*_, where γ_0_ and γ_1_ denote the regression coefficients. The coefficient of correlation *R* quantifies the strength and direction of the linear association between *P* and *D*_*i*_.^48^

#### Ridge Regression

Ridge regression is a regularization technique used to address multicollinearity among predictor variables by introducing an l _2_-norm penalty to the ordinary least-squares (OLS) objective function. The optimization problem is formulated as

$$\min_{\gamma_{0},\gamma }\left\{\sum_{i=1}^{m}{\left(P_{i}-\gamma_{0}-\sum_{j=1}^{k}\gamma_{j}D_{ij}\right)}^{2}+\alpha \sum_{j=1}^{k}\gamma_{j}^{2}\right\}$$

where *P*_*i*_ is the target property for the *i*th observation, *D*_*ij*_ is the value of the *j*th predictor, γ_*j*_ are the regression coefficients, and α ≥ 0 is the regularization parameter controlling the shrinkage. The penalty term $\alpha \sum_{j=1}^{k}\gamma_{j}^{2}=\alpha ∥\gamma {∥}_{2}^{2}$ reduces the magnitude of the regression coefficients, stabilizing estimates and mitigating overfitting caused by strong correlations among predictors.^48^

#### Random Forest Regression

This ensemble method constructs multiple decision trees, each trained on a bootstrap sample of the data with a random subset of features considered for splits. The final prediction is the averaged output across constituent trees:

$$\mathrm{P̂}=\frac{1}{N_{t}}\sum_{t=1}^{N_{t}}h_{t}\left(D\right)$$

where *N*_*t*_ is the number of trees and *h*_*t*_ (*D*) represents the prediction of the *t*-th tree.^48^

#### XGBoost Regression

Extreme gradient boosting employs sequential tree construction, where each subsequent model focuses on residual errors from preceding ensembles. The objective function combines both predictive accuracy and complexity regularization:

$$X=\sum_{i=1}^{m}l_{f}\left(P_{i},{\mathrm{P̂}}_{i}\right)+\sum_{t=1}^{T}\Psi \left(h_{t}\right)$$

where *l*_*f*_ denotes the loss function and Ψ penalizes model complexity.^49^

It should be emphasized that the physicochemical properties considered in this study can also be viewed as higher-level descriptors derived from the molecular structure. Therefore, the present work does not aim at replacing experimental or computational estimation methods but rather at analyzing the extent to which simpler graph-based descriptors can approximate these properties. This perspective allows for a better understanding of descriptor redundancy, interdependence, and model limitations. Although random forest and XGBoost are widely regarded as powerful nonlinear learning algorithms, their performance depends strongly on the availability of sufficiently large and diverse training data sets. Therefore, they were included primarily as benchmark machine-learning regression models against which simpler regression approaches could be objectively evaluated.

### Model Validation

A rigorous validation technique is essential for reliable performance estimation, given the limited sample sizes.

#### Cross-Validation Strategy

Leave-one-out cross-validation (LOOCV) is employed exclusively throughout this study. For data sets comprising *n* compounds, this approach involves iteratively training on *n* – 1 samples and validating on the single excluded sample, repeated *n* times.

#### Hyperparameter Optimization

A comprehensive grid search within an LOOCV is employed to systematically optimize the most influential hyperparameters for each regression model, while secondary parameters are fixed at the default values. The strategy was tailored to each model.^48^

#### Performance Evaluation Metrics

The root mean squared error (RMSE) measures prediction error with sensitivity to larger deviations:

$$RMSE=\sqrt{\frac{\sum_{k=1}^{n}{\left(\rho_{k}-\vartheta_{k}\right)}^{2}}{n}}$$

where ρ_*k*_ and ϑ_*k*_ are the observed and predicted values for sample *k*, respectively, and *n* is the number of test samples.^48^ The present study is based on a relatively small data set of 18 bipolar disorder drugs, reflecting the limited availability of compounds with complete structural and physicochemical information. Accordingly, leave-one-out cross-validation was adopted to provide a rigorous evaluation framework for model performance despite the limited data set size.

## Results

This section describes the computational techniques and algorithmic procedures employed in this study. Initially, the edge-partitioning approach is used to compute degree- and neighborhood-degree-sum-based bond-additive indices for the bipolar drug data set. Then, the exhaustive model selection algorithm is implemented within an LOOCV to predict physicochemical properties from the resulting topological indices.

### Computation of Degree and Neighborhood-Degree-Sum Bond-Additive Indices

In this section, an edge partitioning approach is applied to calculate bond-additive indices based on the vertex degree and neighborhood-degree sums for the molecular graphs. Tables 3 and 4 present the edge partitions of the molecular graphs based on the vertex degrees and neighborhood-degree-sums, respectively.

**Table 3:** Degree-Based Edge Partitions of Bipolar Disorder Drug Graphs

BDs	ς_12_	ς_13_	ς_14_	ς_22_	ς_23_	ς_24_	ς_33_	ς_34_
BD_1_	-	3	-	10	16	-	4	-
BD_2_	1	4	-	20	17	-	6	-
BD_3_	-	2	-	4	12	-	5	-
BD_4_	-	2	-	7	6	-	5	-
BD_5_	-	5	-	7	14	-	4	-
BD_6_	-	2	-	6	14	-	4	-
BD_7_	-	3	1	8	12	2	1	1
BD_8_	-	4	-	3	6	-	4	-
BD_9_	-	3	-	8	14	–	8	-
BD_10_	-	2	-	10	18	-	11	-
BD_11_	-	2	-	5	14	-	4	-
BD_12_	-	3	-	6	6	-	6	-
BD_13_	-	4	-	7	16	-	8	-
BD_14_	-	1	-	9	14	-	3	-
BD_15_	1	-	-	12	13	-	4	-
BD_16_	-	3	-	8	16	-	7	-
BD_17_	2	2	-	2	2	-	1	-
BD_18_	-	2	-	7	18	-	5	-

**Table 4:** Neighborhood-Degree-Sum-Based Edge Partitions of Bipolar Disorder Drug Graphs

BDs	ς_23_^*^	ς_34_^*^	ς_35_^*^	ς_36_^*^	ς_37_^*^	ς_44_^*^	ς_45_^*^	ς_48_^*^	ς_55_^*^	ς_56_^*^	ς_57_^*^	ς_58_^*^	ς_59_^*^	ς_66_^*^	ς_67_^*^	ς_68_^*^	ς_77_^*^	ς_78_^*^	ς_79_^*^	ς_88_^*^	ς_89_^*^	ς_99_^*^
BD_1_	-	-	1	1	1	2	4	-	5	7	4	1	-	1	3	-	1	2	-	-	-	-
BD_2_	1	1	1	2	1	9	5	-	7	7	4	2	-	1	1	2	-	3	1	-	-	-
BD_3_	-	-	2	-	-	1	2	-	2	3	2	1	-	-	2	3	-	2	-	3	-	-
BD_4_	-	-	2	-	-	2	4	-	1	-	4	2	1	-	-	-	-	2	-	-	2	-
BD_5_	-	-	2	2	1	-	2	-	5	10	2	1	-	2	1	-	-	2	-	-	-	-
BD_6_	-	-	2	-	-	1	2	-	6	1	4	1	-	-	4	1	1	2	-	1	-	-
BD_7_	-	-	2	1	-	-	2	1	8	6	2	2	-	-	1	2	-	-	-	1	-	-
BD_8_	-	-	1	2	1	-	2	-	2	2	-	2	-	1	1	1	-	1	-	1	-	-
BD_9_	-	-	1	2	-	-	4	-	6	4	2	4	-	1	1	2	-	-	-	3	3	-
BD_10_	-	-	-	-	2	2	4	-	4	2	8	2	-	1	4	1	1	4	4	1	-	1
BD_11_	-	-	2	-	-	1	2	-	4	2	4	-	-	-	4	2	1	2	-	1	-	-
BD_12_	-	-	2	1	–	2	4	-	-	-	1	3	1	1	1	1	-	1	-	1	2	-
BD_13_	-	-	1	2	1	-	2	-	6	5	3	4	-	1	1	3	-	4	-	2	-	-
BD_14_	-	-	1	-	-	1	4	-	6	2	7	1	-	1	1	-	1	2	-	-	-	-
BD_15_	1	1	-	-	-	4	5	-	2	3	5	1	-	-	3	1	1	2	-	1	-	-
BD_16_	-	-	1	1	1	1	2	-	6	4	4	4	-	1	2	1	-	5	-	1	-	-
BD_17_	2	-	4	-	-	-	-	-	-	-	3	-	-	-	-	-	-	-	-	-	-	-
BD_18_	-	-	1	1	-	1	2	-	4	5	5	2	-	1	6	-	1	2	-	1	-	-

To illustrate the calculation, consider valproic acid (BD_17_), and from Table 3, the edge-partition entries for BD_17_ are ς_12_ = 2, ς_13_ = 2, ς_22_ = 2, ς_23_ = 2, and ς_33_ = 1. For calculating the first Zagreb degree-based index (*M*_1_),

$$M_{1}\left({BD}_{17}\right)=\varsigma_{12}\left(1+2\right)+\varsigma_{13}\left(1+3\right)+\varsigma_{22}\left(2+2\right)+\varsigma_{23}\left(2+3\right)+\varsigma_{33}\left(3+3\right)=38$$

For the first Zagreb neighborhood-degree-sum-based index of the BD_17_ graph from Table 4, the entries are $\varsigma_{23}^{*}=2,\varsigma_{35}^{*}=4,\varsigma_{57}^{*}=3$ , and hence, the computation of the first Zagreb neighborhood-degree-sum-based index (*M*_1_^*^) is given by

$$M_{1}^{*}\left({BD}_{17}\right)=\varsigma_{23}^{*}\left(2+3\right)+\varsigma_{35}^{*}\left(3+5\right)+\varsigma_{57}^{*}\left(5+7\right)=78$$

The computed values of the degree-based and neighborhood-degree-sum-based topological indices are reported in Tables 5 and 6.

**Table 5:** Calculated Values of Degree-Based Topological Indices

BDs	*M*_1_	*M*_2_	*F*	*SDD*	*ISI*	*H*	*SC*	*R*	σ	*SO*	*ABC*	*ABS*	*AZ*
BD_1_	156	181	390	72.6667	37.45	14.2333	15.2884	14.5974	28	112.4305	23.5009	24.8519	263.6875
BD_2_	220	250	534	104.6667	53.0667	21.4667	22.6295	21.9567	34	158.2039	34.1360	35.6150	385.8438
BD_3_	114	139	298	50.6667	27.4	9.4667	10.4078	9.7203	20	82.1181	16.2800	17.6203	191.7031
BD_4_	96	115	244	43.6667	23.2	8.5667	9.2245	8.7709	14	68.9701	14.1587	15.0940	167.7031
BD_5_	142	163	360	69	33.55	12.9333	13.8940	13.4356	34	103.0587	21.5984	22.5956	230.4375
BD_6_	126	150	322	57	30.3	10.9333	11.8940	11.2035	22	90.7434	18.4418	19.7672	212.3125
BD_7_	134	154	350	65.3333	31.331	11.9857	12.9165	12.4601	42	97.6909	20.1840	21.1426	213.7100
BD_8_	82	96	214	40.3333	19.2	7.2333	7.8163	7.5922	22	59.7383	12.2966	12.8633	131.0625
BD_9_	162	197	420	72.3333	39.05	13.7667	15.0270	14.1142	26	116.5331	23.3392	25.1545	277.2500
BD_10_	204	253	532	87.6667	49.6	16.8667	18.5406	17.1698	26	146.1778	28.7653	31.4095	356.0469
BD_11_	122	146	314	55	29.3	10.4333	11.3940	10.7035	22	87.9150	17.7347	19.0601	204.3125
BD_12_	102	123	264	47	24.45	8.9000	9.6328	9.1815	18	73.5465	14.9348	15.9105	174.4688
BD_13_	172	208	448	78	41.2	14.5667	15.9214	15.0080	32	124.0780	24.8628	26.7037	288.6250
BD_14_	128	150	318	57.6667	31.05	11.6000	12.4857	11.7928	18	91.8238	19.0800	20.3649	221.5469
BD_15_	140	164	342	62.6667	34.2667	13.2000	14.0241	13.3477	14	100.0199	21.0514	22.3984	253.5625
BD_16_	166	200	428	74.6667	39.95	14.2333	15.5132	14.5974	28	119.5016	24.0867	25.8872	281.8594
BD_17_	38	39	90	22	8.7333	4.4667	4.4574	4.7187	12	27.9073	6.5423	6.3488	66.1406
BD_18_	156	187	400	69.6667	37.6	13.3667	14.5911	13.6698	26	112.2367	22.6440	24.3892	263.7031

**Table 6:** Calculated Values of Neighborhood-Degree-Sum-Based Topological Indices

BDs	*M*_1_^*^	*M*_2_^*^	F*	SDD*	ISI*	H*	SC*	R*	σ*	SO*	ABC*	ABS*	AZ*
BD_1_	362	1004	2078	68.7512	88.8574	6.1921	10.0717	6.2585	70	258.2552	18.3103	29.7375	1254.967
BD_2_	500	1345	2788	99.9131	122.6997	9.7196	15.1690	9.8187	98	356.7515	27.2516	42.8444	1683.034
BD_3_	278	868	1782	47.5202	68.5004	4.0050	6.7419	4.0414	46	197.9770	12.3188	20.8953	1152.637
BD_4_	230	688	1442	42.0595	56.1109	3.7081	6.0419	3.7552	66	164.5756	11.0016	18.0438	894.4435
BD_5_	326	888	1850	63.2417	79.6749	5.6481	9.1787	5.7300	74	233.0472	16.7411	27.0247	1095.628
BD_6_	300	887	1820	53.5631	73.9836	4.6895	7.7686	4.7271	46	213.5566	14.1381	23.5328	1146.727
BD_7_	308	852	1780	58.7024	75.2998	5.2298	8.5286	5.2938	76	220.1565	15.5399	25.2457	1055.146
BD_8_	192	548	1162	36.7702	46.4205	3.1319	5.1348	3.2003	66	137.9487	9.4441	15.3507	687.4309
BD_9_	394	1213	2512	68.9607	96.6078	5.7935	9.7219	5.8630	86	281.2386	17.8090	29.9561	1600.962
BD_10_	506	1596	3308	85.6587	124.0656	6.9308	11.8560	7.0101	116	361.1813	21.8028	37.3657	2113.620
BD_11_	292	875	1792	51.4548	72.0625	4.4604	7.4275	4.4959	42	207.7908	13.5300	22.6544	1138.645
BD_12_	246	750	1576	44.5310	59.8639	3.8392	6.2954	3.8986	76	176.2281	11.5050	18.9756	982.3065
BD_13_	416	1251	2612	73.8833	101.5633	6.0958	10.2841	6.1860	110	297.5428	18.9155	31.8003	1608.743
BD_14_	300	845	1740	55.6179	73.9095	5.0031	8.1886	5.0402	50	213.6621	14.8642	24.3658	1063.543
BD_15_	328	936	1920	61.5869	80.9550	5.8684	9.2967	5.9083	48	233.3992	16.6932	26.8887	1203.376
BD_16_	400	1192	2482	71.3393	97.8597	5.9849	10.0425	6.0610	98	285.8195	18.4169	30.8565	1528.605
BD_17_	78	177	384	19.7429	18.6500	2.3000	3.1747	2.3564	30	56.3419	5.5476	7.7519	207.125
BD_18_	374	1107	2280	66.2333	92.0443	5.6411	9.4641	5.6930	66	266.4934	17.2983	29.0375	1420.160

### Algorithm

Algorithm 1 performs exhaustive model selection for predicting physicochemical properties from topological indices using the LOOCV. For each property, it evaluates four model families, which are linear, ridge, random forest, and XGBoost, based on minimizing the LOOCV and RMSE. Linear models are fitted on individual indices, while ridge regression explores all subsets of up to two indices with a grid search over α∈A . Tree-based models are trained on the complete feature matrix using predefined hyperparameter grids ( $H_{RF}$ and $H_{XGB}$ ), with only the configuration achieving the lowest LOOCV RMSE retained as the optimal model. The algorithm outputs, for each property, the best-performing model, selected indices or hyperparameters, and the corresponding LOOCV metric.

The objective of evaluating multiple model families was not to identify the most sophisticated learning algorithm but rather to determine the appropriate level of model complexity for graph-theoretical descriptors derived from a small molecular data set. For each physicochemical property, the exhaustive search evaluated 13 univariate linear models and 91 two-descriptor ridge regression models, together with all predefined random forest and XGBoost hyperparameter configurations under leave-one-out cross-validation. Although this comprehensive search increases the possibility of model-selection bias, LOOCV was consistently applied to every candidate model to reduce optimistic performance estimation. Owing to the limited sample size, external validation, applicability domain analysis, and Y-randomization were not performed and remain important directions for future work.

### Statistical Analysis and QSPR Predictive Models

Algorithm 1 is applied to identify QSPR models that relate physicochemical properties of bipolar disorder drugs to degree-based and neighborhood-degree-sum-based topological indices. The search is deliberately constrained to one- and two-index models to preserve interpretability while using LOOCV to provide reliable small-sample estimates of predictive performance. Tables 7 and 8 present the RMSE values of the indices based on the degree and neighborhood-degree-sum, respectively. These results facilitate a direct comparison of the predictive performance and explanatory power across the indices and model classes. Under LOOCV, the models with the lowest RMSE are obtained using algorithm 1 with the comprehensive set of degree-based indices.

**Table 7:** Models with the Lowest RMSE of Degree-Based Indices

model	BP	EoV	FP	MR	POL	MV	MW	COM	HAC
linear	53.22729	6.25032	29.97454	5.70331	2.00184	37.22389	19.5727	48.01924	0.2227
ridge	**52.64356**	**5.73529**	**28.90501**	**5.40792**	**1.89624**	**24.52985**	**15.72806**	**46.55291**	**0.18183**
RF	77.1282	8.59615	43.5821	14.29568	5.7424	54.92338	55.06486	74.81836	3.40198
XGB	91.76775	9.9083	50.66741	15.08319	6.04311	55.88146	60.15807	68.73905	3.44789

**Table 8:** Models with the Lowest RMSE of Neighborhood-Degree-Sum-Based Indices

model	BP	EoV	FP	MR	POL	MV	MW	COM	HAC
linear	55.68283	6.38721	30.95955	5.73901	2.00294	26.18349	20.67326	47.34495	0.37696
ridge	**53.14273**	**5.72658**	**28.71509**	**5.30584**	**1.78188**	**21.68524**	**15.36232**	**44.81001**	**0.29034**
RF	86.28882	9.54371	47.80099	15.86865	6.29466	56.74909	59.99278	72.10821	3.63809
XGB	97.76274	10.74073	54.10157	18.46733	7.35454	65.10074	68.34961	73.88009	4.36712

The comparative analysis reveals consistent and interpretable trends across all of the evaluated model families. Univariate linear and bivariate ridge regression models demonstrated stable and reproducible predictive performance across all investigated physicochemical properties, indicating reliable generalization under LOOCV. In contrast, although random forest and XGBoost are capable of modeling complex nonlinear relationships, their predictive performance deteriorated under LOOCV because of the limited sample size, suggesting an increased susceptibility to overfitting. These findings demonstrate that regularized linear models provide reliable and interpretable QSPR predictions for small molecular data sets. Tables 9 and 10 illustrate the best model, predictive indices, MSE, RMSE, and *R* ^2^ values for the degree-based and neighborhood-degree-sum-based indices, respectively.

**Table 9:** Optimal Degree-Based Models Using Exhaustive Selection with LOOCV

property	model	indices	MSE	RMSE	*R* ^2^
**BP**	**ridge**	*SDD*, *AZ*	**2771.34424**	**52.64356**	**0.80559**
EoV	ridge	*M*_2_, *SDD*	32.89351	5.73529	0.83784
FP	ridge	*ABC*, *AZ*	835.49982	28.90501	0.82422
MR	ridge	*R*, *AZ*	29.24562	5.40792	0.97029
POL	ridge	*M*_2_, *R*	3.59574	1.89624	0.97589
MV	ridge	*M*_2_, *R*	601.71363	24.52985	0.9325
MW	ridge	*R*, *AZ*	247.37188	15.72806	0.98027
COM	ridge	*F*, *AZ*	2167.17332	46.55291	0.94226
**HAC**	**ridge**	*R*, σ	**0.03306**	**0.18183**	**0.99943**

**Table 10:** Optimal Neighborhood-Degree-Sum-Based Models Using Exhaustive Selection with LOOCV

property	model	indices	MSE	RMSE	*R* ^2^
BP	ridge	SO*, AZ*	2824.14948	53.14273	0.80188
**EoV**	**ridge**	SO*, AZ*	**32.79377**	**5.72658**	**0.83833**
**FP**	**ridge**	SO*, AZ*	**824.55624**	**28.71509**	**0.82652**
**MR**	**ridge**	SC*, H*	**28.15195**	**5.30584**	**0.9714**
**POL**	**ridge**	SC*, H*	**3.1751**	**1.78188**	**0.97871**
**MV**	**ridge**	R*, ISI*	**470.2498**	**21.68524**	**0.94725**
**MW**	**ridge**	F*, SDD*	**236.00097**	**15.36232**	**0.98118**
**COM**	**ridge**	ABS*, σ*	**2007.93705**	**44.81001**	**0.9465**
HAC	ridge	R*, SDD*	0.0843	0.29034	0.99855

Table 11 reports the in-sample regression statistics and equations corresponding exclusively to the optimal LOOCV models listed in Tables 9 and 10. No alternative models are introduced in this table, ensuring consistency between model selection and regression analysis, and the corresponding regression equations for the properties with optimal models are outlined below:

- BP = 5.5172­(*SDD*) + 0.0722­(*AZ*) + 159.021.
- EoV = 0.4233­(SO*) – 0.0468­(AZ*) + 39.9646
- FP = 1.9667­(SO*) – 0.2096­(AZ*) + 70.5049
- MR = 6.0835­(SC*) + 9.4298­(H*) – 0.971.
- POL = 2.3151­(SC*) + 3.7793­(H*) + 0.4174.
- MV = 71.4322­(R*) – 1.0325­(ISI*) – 10.3471
- MW = 8.6927­(SDD*) – 0.0794­(F*) + 4.6157
- COM = 19.3882­(ABS*) + 1.9622­(σ*) – 135.4188
- HAC = 1.9881­(*R*) + 0.0305­(σ) + 0.1237

**Table 11:** Regression Results for QSPR Models

property	*R*	*R* ^2^	adj. *R* ^2^	MSE	RMSE
BP	0.9214	0.84898	0.83954	2164.41924	46.52332
EoV	0.93854	0.88086	0.87342	24.52341	4.95211
FP	0.93755	0.87899	0.87143	581.93442	24.12332
MR	0.9881	0.97634	0.97486	23.29499	4.82649
POL	0.99098	0.98205	0.98093	2.67868	1.63667
MV	0.98266	0.96562	0.96347	307.26858	17.52908
MW	0.99315	0.98634	0.98548	173.35759	13.16653
COM	0.98151	0.96336	0.96107	1375.68861	37.09028
HAC	0.99992	0.99984	0.99983	0.00935	0.0967

Table 11 indicates that most molecular properties are best predicted using neighborhood-degree-sum-based indices, whereas the boiling point and heavy atom count are more accurately described by degree-based indices. This observation suggests that neighborhood-degree-sum-based descriptors encode richer local structural information by incorporating the connectivity of adjacent atoms, thereby capturing subtle structural variations that influence the most physicochemical properties. Conversely, degree-based descriptors appear sufficient for properties such as boiling point and heavy atom count, whose dependence on molecular topology is comparatively simpler. Figures 2 and 3 present a graphical comparison of the actual and estimated values for the properties of drugs used to treat bipolar disorder.

**Figure 2:**
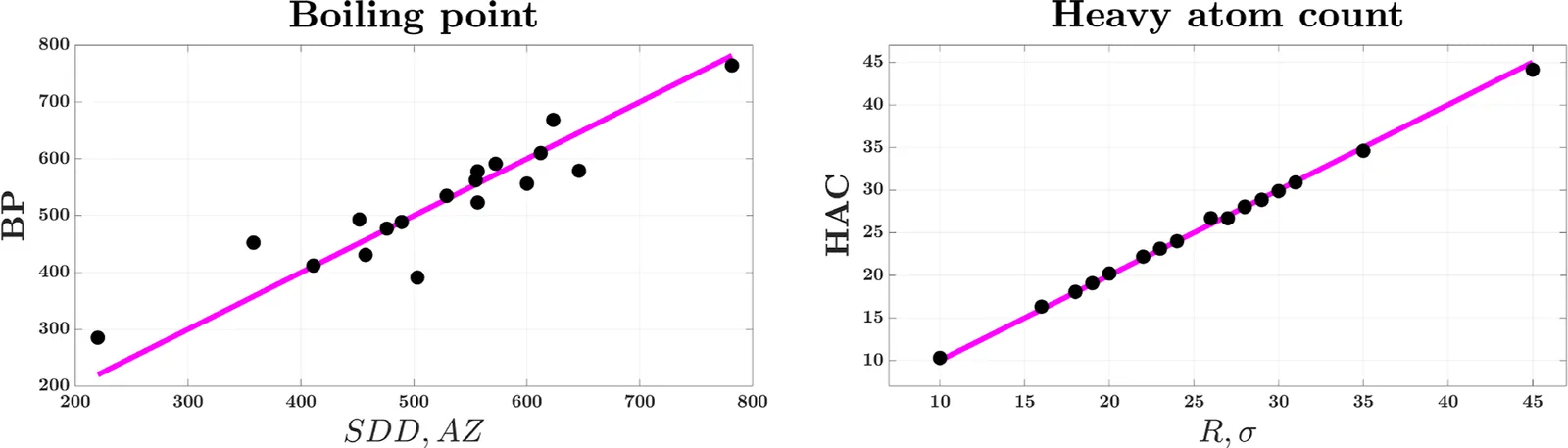
Regression plots based on degree-based indices.

**Figure 3:**
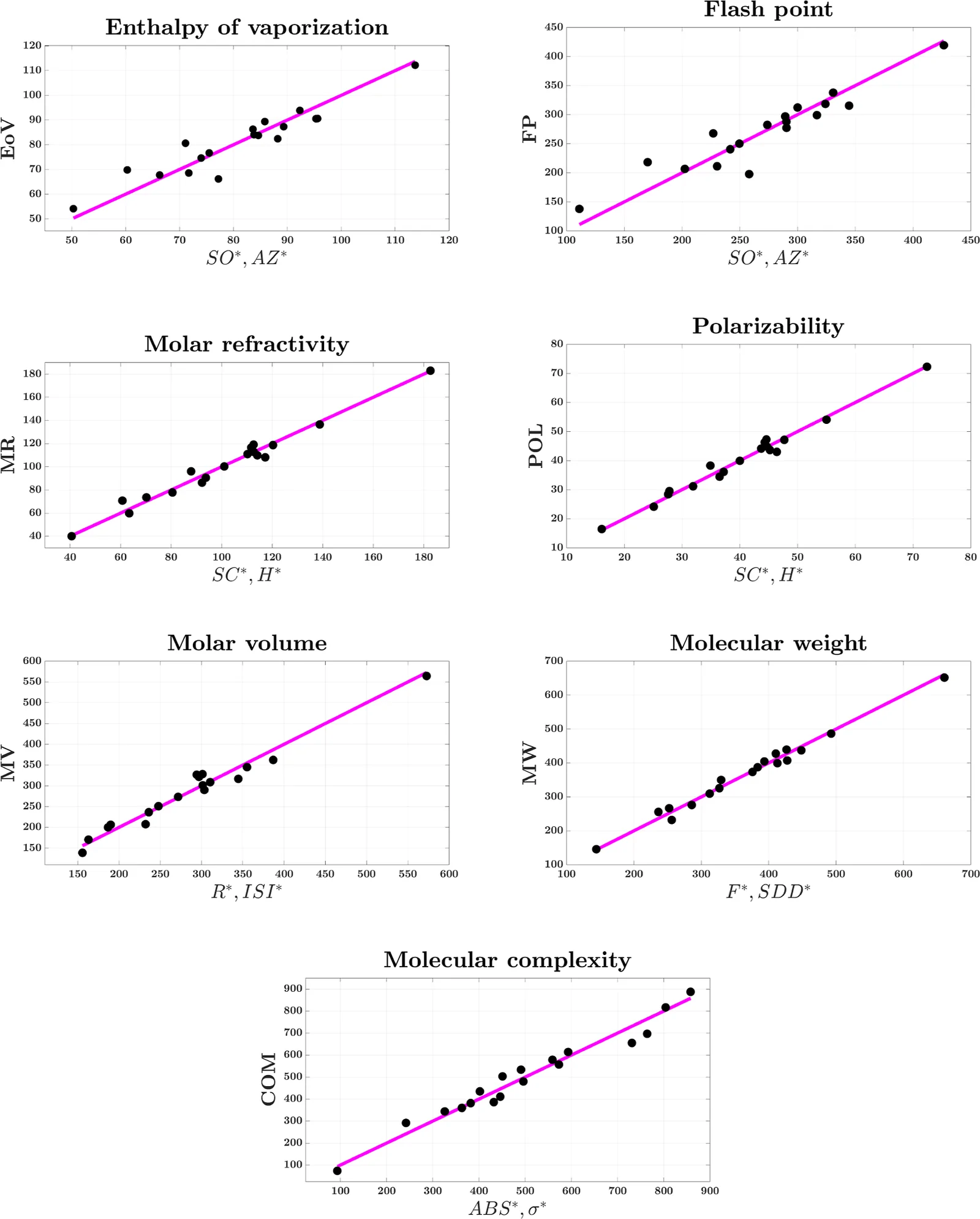
Regression plots based on neighborhood-degree-sum-based indices.

The inclusion of random forest and XGBoost was intended to evaluate whether nonlinear machine-learning algorithms provide additional predictive capability beyond interpretable regression models. Across all physicochemical properties, neither ensemble method produced lower LOOCV RMSE values than ridge regression. Rather than indicating a limitation of these algorithms, this outcome reflects the extremely small sample size (*n* = 18), where high-capacity learners are susceptible to an increased variance and overfitting. Consequently, the comparative analysis suggests that simpler regularized regression models remain the preferred choice for exploratory QSPR studies involving limited molecular data sets.

## Discussion

The comparative evaluation demonstrates that increasing algorithmic complexity did not improve predictive performance for the present data set. Although random forest and XGBoost are capable of modeling highly nonlinear relationships, their LOOCV prediction errors remained consistently larger than those obtained by ridge regression. This behavior is consistent with the well-recognized tendency of ensemble tree methods to exhibit higher prediction variance when trained on extremely small data sets such as the present one (*n* = 18). These findings suggest that regularized linear models provide a more appropriate balance between predictive performance and interpretability for exploratory QSPR studies involving limited molecular data sets. Molecular weight and heavy atom count differ from the remaining physicochemical properties because both are strongly related to molecular size. Since hydrogen atoms are omitted in molecular graphs, these end points are inherently correlated with graph size and connectivity, making them comparatively easier to predict using graph-theoretical descriptors. Consequently, their excellent predictive performance should be interpreted primarily as reflecting descriptor consistency with molecular size rather than the superior modeling of more complex physicochemical properties. The present study focuses specifically on assessing the predictive capability of graph theoretical topological indices. Consequently, comprehensive descriptor libraries such as RDKit and Mordred were not included, as incorporating heterogeneous descriptor families would prevent an isolated evaluation of the proposed graph-theoretical descriptors. A systematic comparison with these descriptor libraries represents an important direction for future research using larger and more diverse molecular data sets. From a practical perspective, the proposed framework provides a computationally inexpensive and interpretable approach for preliminary molecular property prediction during the early stages of drug discovery. Such graph-based QSPR models may assist in prioritizing compounds for more detailed computational or experimental investigations, thereby reducing the overall cost and time associated with the molecular screening.

## Conclusion

This study investigated the effectiveness of degree-based and neighborhood-degree-sum-based topological indices for the QSPR modeling of drugs used to treat bipolar disorder. Graph-theoretical descriptors were computed and evaluated by using interpretable regression and machine learning regression models to predict selected physicochemical properties. Predictive models based on linear regression, ridge regression, random forest, and XGBoost were comparatively evaluated to determine the level of model complexity most appropriate for an interpretable QSPR analysis of a small molecular data set. The results showed that bivariate ridge regression models outperformed univariate linear and nonlinear tree-based approaches, achieving correlation coefficients greater than 0.9214, demonstrating strong relationships between molecular topology and the investigated physicochemical properties. Degree-based descriptors produced the most accurate models for boiling point and heavy atom count, whereas neighborhood-degree-sum descriptors achieved lower prediction errors for the remaining seven physicochemical properties, highlighting their stronger ability to capture local structural characteristics. The comparative evaluation of classical regression and modern machine-learning algorithms indicates that, for small graph-theoretical data sets, parsimonious regression models provide more reliable predictive performance than complex ensemble learners. Nevertheless, random forest and XGBoost remain valuable benchmark models for comparison with interpretable regression approaches. Future research will focus on extending the methodology to larger and more structurally diverse molecular data sets, performing external validation and applicability-domain analysis, integrating graph-theoretical descriptors with complementary molecular and quantum-chemical descriptors, and investigating explainable machine learning techniques to further improve predictive performance and model interpretability.
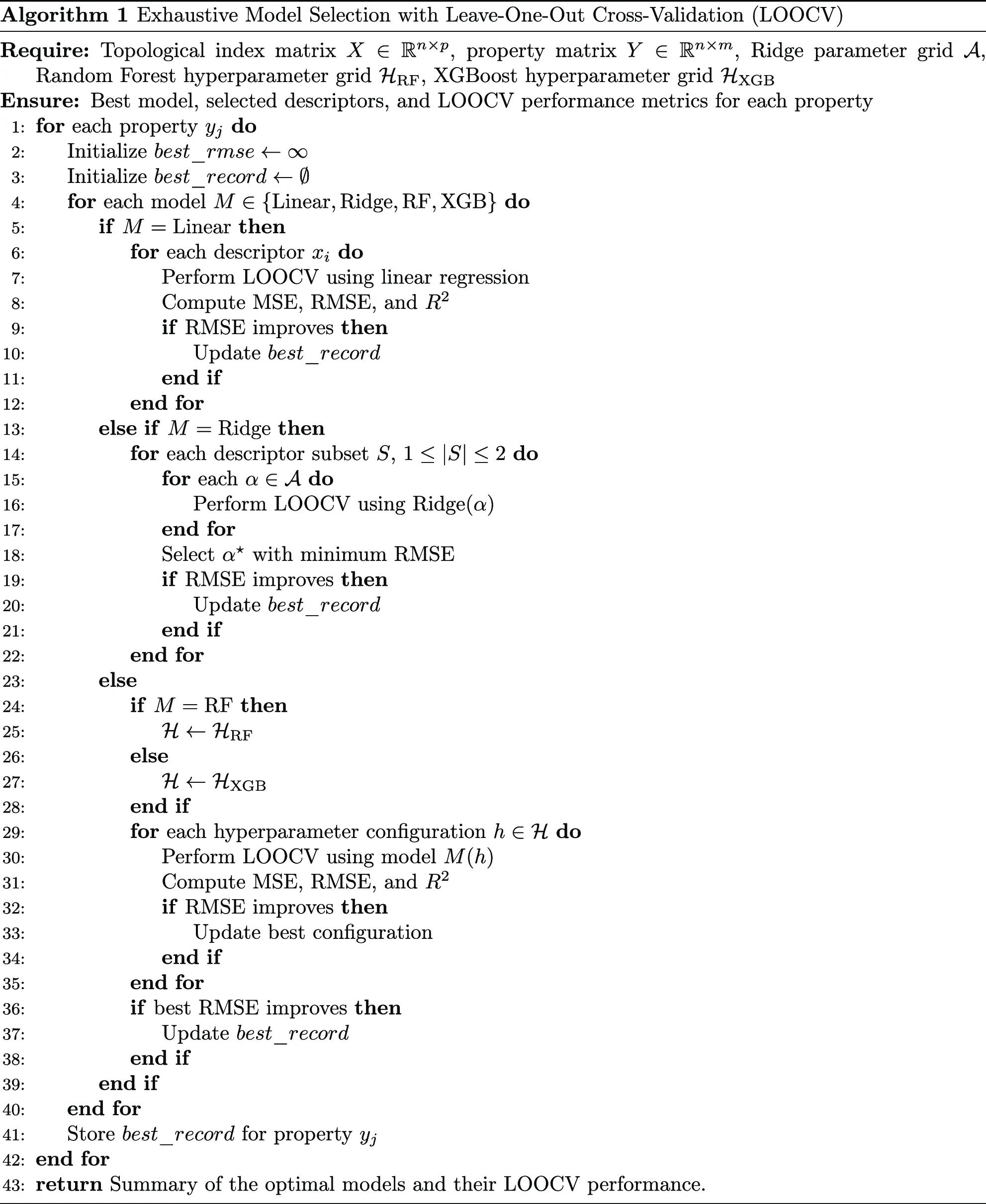


## Data Availability

The data sets and source code used in this study are available from the corresponding author upon reasonable request.
